# Rare and Underestimated Association of Pulmonary Embolism and Olanzapine Therapy; Report of Two Cases

**DOI:** 10.22037/aaem.v9i1.1063

**Published:** 2021-02-05

**Authors:** Hammam Rasras, Mustapha Beghi, Maryem Samti, Nabila Ismaili, Noha El Ouafi

**Affiliations:** 1 Department of Cardiology, Mohammed VI University Hospital of Oujda, Mohammed First University of Oujda, Morocco.; 2 Laboratory of Epidemiology, Clinical Research and Public Health, Faculty of Medicine and Pharmacy, Mohammed the First University of Oujda, Morocco.

**Keywords:** Pulmonary embolism, venous thromboembolism, risk factors, antipsychotic agents, olanzapine

## Abstract

Venous thromboembolic disease (VTD) is a very common and severe pathological condition in which there are many predisposing factors. Olanzapine is a drug frequently used in psychiatric practises; it is thought to increase the risk of VTD. Here, we report two cases, a young man and a woman, with a medical history of schizophrenia treated by olanzapine who developed pulmonary embolism and we did not find any aetiologies of VTD in them. Due to the link between olanzapine and pulmonary embolism, which has been previously described, olanzapine is considered responsible for this problem. Two mechanisms have been reported in the literature in this regard; significant weight gain and lethargy, which are very common side effects of olanzapine. So far, no direct effect of olanzapine on platelet aggregation or coagulation has been found. In patients developing VTD while being treated with olanzapine, discontinuation of olanzapine as a treatment option must be done with an adjustment of antipsychotic treatment and regular monitoring of psychic symptoms. Since the diagnosis of pulmonary embolism is not easy to make in a schizophrenic patient, clinicians should take that in consideration when prescribing these drugs and when facing clinical situations where VTD is suspected.

## Introduction

Venous thromboembolic disease (VTD) is a multifactorial disease, which is represented by two entities: deep venous thrombosis (DVT) or pulmonary embolism (PE). VTD usually develops due to many thromboembolic risk factors, such as old age, context of immobilization, post-surgery or pregnancy, past history of DVT, neoplasia, certain drugs (oestrogens, chemotherapy), antiphospholipid syndrome, obesity, sedentarily, and hypercoagulability states, including mutation of factor V Leiden or prothrombin (1). Like many diseases, VTD has predisposing factors, which are grouped into three categories, termed the Virchow's triad: endothelial injury, stasis of blood flow, and, hypercoagulability (2). It is a major cause of morbidity and mortality among hospitalized patients, with an annual incidence of 1 per 1000 population (3). Olanzapine is an atypical antipsychotic (AAP) (second-generation antipsychotic), prescribed for schizophrenic patients, especially those with positive symptoms. This therapeutic class has several side effects, including metabolic syndrome and diabetes, hyperprolactinemia, sexual dysfunction, and weight gain (4), which is more frequently observed in olanzapine. The incidence of VTD in AAP users is increased by 2.20 (95% CI: 1.22-3.95) times (3).

We report two cases, a young man and a woman with a history of paranoid schizophrenia, who presented with PE while using olanzapine without inherited risk factors for VTD. We further explored the possible association between olanzapine and VTD. Lastly, we took a brief look at and reviewed the potential mechanisms behind this association.

## Cases presentation:

**Case 1:**

**Figure 1:**
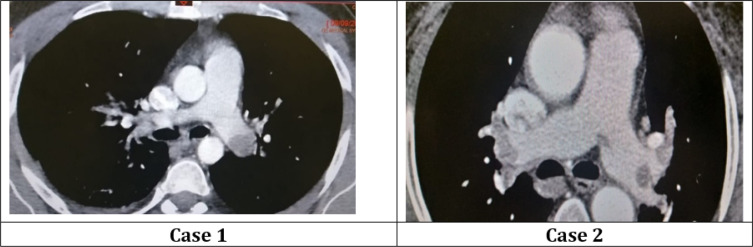
Axial cut of thoracic computed tomography angiography showing embolism in right and left pulmonary artery

A 28-year old male patient, with no risk factors for VTD, with a medical history of schizophrenia on olanzapine therapy, was admitted for an acute onset of hallucinations and delirious behaviour with dyspnoea. On admission, he was confused, afebrile, had tachycardia with a blood pressure at 130/50mmHg and normal oxygen saturation in room air. Cardiovascular, respiratory, and abdominal examinations’ findings were insignificant. There were no signs of meningism. Blood tests revealed high D-dimer (7.11µg/l), troponin (2928 ng/l) and B-type natriuretic peptide (BNP) (2415 pg/m) levels. Chest X-ray showed an elevation of the right dome of diaphragm with low abundance pleurisy, whilst his electrocardiogram (ECG) showed sinus tachycardia and right bundle branch block with a right axis deviation. Computed tomography (CT) pulmonary angiogram revealed an extensive right and left pulmonary embolism (Figure 1). Systolic pulmonary arterial pressure (sPAP) was 41 mmHg, paradoxical septum, and dilated right ventricle (RVD/VG = 1.14) with systolic dysfunction were observed in the transthoracic echocardiography (TTE). The simplified pulmonary embolism severity index (s-PESI) was 1 and the patient was classified as a high intermediate risk patient. In etiological assessment: doppler ultrasound of two legs, tests for thrombophilia (no family history of thrombophilia was found), tumor markers, and thoracic, abdominal, and pelvic CT angiography showed no abnormalities. All of the diagnostic tests were normal, and since the only possible risk factor was olanzapine therapy, it was terminated after psychiatric consultation, and then he was put on aripiprazole. Then, he was treated with anticoagulants.

**Case 2:**

A 68-year old female patient, obese with a medical history of psychotic depression on olanzapine therapy, was admitted for an atypical chest pain and dyspnoea. On admission, she was asthenic, had no fever, heart rate was 111 beats/minute, blood pressure was 100/50 mmHg, and respiratory rate was 28 cycles/minute. Her oxygen saturation level was low (90 % in room air). Physical examination findings were insignificant. Blood tests revealed high D-dimer (1.88 µg/l), troponin (7439 ng/l) and BNP (1324 pg/m) levels. Chest X-ray was normal, ECG showed sinus tachycardia. CT pulmonary angiography revealed a bilateral extensive pulmonary embolism (Figure 1). TTE showed a dilated right ventricle with systolic dysfunction and a systolic pulmonary arterial pressure (sPAP) at 64 mmHg. The s-PESI was 2 and the patient was classified as a high intermediate risk patient. In etiological assessment: doppler ultrasound of two legs, tumor markers, gynaecological examination, mammography, and thoracic, abdominal and pelvic CT angiography revealed no anomalies. Then, she was put on heparin therapy with relay by vitamin K antagonist until the repeated international normalized ratio (INR) was between 2 and 3. Heparin was stopped on the 9th day, with good evolution.

## Discussion

VTD is a complex disease, involving interaction between acquired or inherited predispositions to thrombosis (Thrombophilia) and environmental exposures (clinical risk factors). Many studies have considered olanzapine as a risk factor that might increase the risk of VTD. This presentation was reported for the first time in Germany in 1950s (4). The underlying mechanism is still unknown. In our study, we are trying to take a brief look at literature and review the hypotheses of the potential mechanisms behind this association.

Some clinical and pharmacological studies have been published in this domain, trying to explain the causality of this association between olanzapine and VTD. In 2002, Hägg et al. (5) found an increased level of antiphospholipid antibodies and hyperprolactinemia in patients on olanzapine therapy, and then considered it as an independent risk factor for thromboembolic events. In 2008, Kannan et al. reported six cases of fatal PE in six patients who were on olanzapine therapy for a long period; they showed that olanzapine binds to serotonin receptors, and then activates serotonergic system, which stimulates platelet aggregation; it can also bind to alpha-receptors, which contributes to the formation thrombosis by causing hypotension and venous blood stasis (1). Some hypotheses were proposed by these two teams and it was suggested that olanzapine can induce hyperleptinemia, which might lead to abnormalities in fibrinolysis, as well as metabolic disorders, such as dyslipidaemia and hyper-homocysteinemia, which play an important role in thrombotic events (1, 5). A retrospective study on the WHO database showed that VTD occurred with a higher frequency in patients on olanzapine and clozapine compared to those on other AAPs, and in 60% of cases this event occurred within the first 3 months of treatment. Moreover, like other studies, some plausible mechanisms were suggested; such as antipsychotic-induced sedation, obesity, hyperleptinemia, the presence of antiphospholipid antibodies, and enhanced platelet aggregation (6). In 2018, a study was performed by the Netherlands Pharmacovigilance Centre Lareb on seventeen patients who developed VTD while using olanzapine, and only a few of them had thromboembolism risk factors. This study showed a higher incidence rate of VTD in patients on AAP therapy compared with populations who had predisposing factors of VTD, and that - like other mentioned studies - was explained by the drug’s side effects, causing platelet aggregation and inducing the blockade of histamine-1 receptor and α-1 receptor. It has also been shown that olanzapine and clozapine have the most profound impact compared to other AAPs. Moreover, discontinuation of olanzapine has been suggested for those who develop VTD, with a control of psychotic symptoms and antipsychotic treatment options (3). A pharmacological study performed by Almuqdadi et al. has confirmed that olanzapine could antagonise the serotonin (5-HT2A) receptors; since these receptors are present on platelets, olanzapine is thought to be a factor influencing platelet aggregation (7). Other studies were performed in the same domain trying to explain the mechanism behind this association, for instance, Carrizo et al. found increased leptin and Plasminogen Activator Inhibitor-1 (PAI-1) levels in patients treated with olanzapine, and believed that this makes them more exposed to thromboembolic events (8).

Both the Dutch website for drug information ‘FarmacotherapeutischKompas’ and the Dutch Summary of Product Characteristics (SmPC) indicated that there is no direct relationship between olanzapine and VTD and that the underlying cause of VTD are the side effects induced by olanzapine (9, 10).

In our cases, no risk factor of VTD was found in the first patient and etiological assessment did not reveal any abnormalities for any of the patients, and we believe that olanzapine is the underlying cause of PE, similar to the previously reported cases.

No validated risk assessment score is available for identification of psychiatric patients who might benefit from pharmacological VTD prophylaxis, and we are looking forward to performance of studies that can establish a scoring system for evaluating the risk of thromboembolic events before initiation of therapy with olanzapine or other AAPs.

## Conclusion:

Olanzapine is an increasingly prescribed drug for patients with schizophrenia, and like all other drugs, it has side effects that can be banal or potentially serious and life threatening. The incidence rate of VTD in patients treated with olanzapine is higher than that of the general population, and despite the absence of studies that confirm the direct link between them, olanzapine is considered to be a factor that can increase the risk of thromboembolic events. Further studies, especially pharmacological studies that can confirm the underlying cause and find prophylactic treatments, especially for patients who already have thromboembolic risk factors, should be considered.
